# Genome-Wide Analysis of the Five Phosphate Transporter Families in *Camelina sativa* and Their Expressions in Response to Low-P

**DOI:** 10.3390/ijms21218365

**Published:** 2020-11-07

**Authors:** Dhondup Lhamo, Qiaolin Shao, Renjie Tang, Sheng Luan

**Affiliations:** 1 Department of Plant and Microbial Biology, University of California at Berkeley, Berkeley, CA 94720, USA; qiaolin@berkeley.edu (Q.S.); rjtang@berkeley.edu (R.T.); 2 State Key Laboratory of Crop Genetics and Germplasm Enhancement, College of Agriculture, Nanjing Agricultural University, Nanjing 210095, China

**Keywords:** PHT gene family, phylogenetic analysis, chromosomal distribution, protein domain, *cis*-element, tissue expression, low-P response, *Camelina* (false flax)

## Abstract

Phosphate transporters (PHTs) play pivotal roles in phosphate (Pi) acquisition from the soil and distribution throughout a plant. However, there is no comprehensive genomic analysis of the PHT families in *Camelina sativa*, an emerging oilseed crop. In this study, we identified 73 CsPHT members belonging to the five major PHT families. A whole-genome triplication event was the major driving force for CsPHT expansion, with three homoeologs for each *Arabidopsis* ortholog. In addition, tandem gene duplications on chromosome 11, 18 and 20 further enlarged the CsPHT1 family beyond the ploidy norm. Phylogenetic analysis showed clustering of the CsPHT1 and CsPHT4 family members into four distinct groups, while CsPHT3s and CsPHT5s were clustered into two distinct groups. Promoter analysis revealed widespread *cis*-elements for low-P response (P1BS) specifically in *CsPHT1s*, consistent with their function in Pi acquisition and translocation. In silico RNA-seq analysis revealed more ubiquitous expression of several *CsPHT1* genes in various tissues, whereas *CsPHT2s* and *CsPHT4s* displayed preferential expression in leaves. While several *CsPHT3s* were expressed in germinating seeds, most *CsPHT5s* were expressed in floral and seed organs. Suneson, a popular *Camelina* variety, displayed better tolerance to low-P than another variety, CS-CROO, which could be attributed to the higher expression of several *CsPHT1/3/4/5* family genes in shoots and roots. This study represents the first effort in characterizing CsPHT transporters in *Camelina*, a promising polyploid oilseed crop that is highly tolerant to abiotic stress and low-nutrient status, and may populate marginal soils for biofuel production.

## 1. Introduction

Phosphorus (P) is an essential macronutrient in all organisms, which provides a backbone to nucleic acids, a polarity to cell membranes, and participates in energy transfer and signal transduction processes [1,2]. Plants acquire P in the form of inorganic phosphate (Pi), which is present at a very low level (<10 µM) in the soil due to its uneven distribution and low solubility [3,4]. Therefore, application of fertilizers has become a common practice in agriculture to ensure crop productivity [2,3]. Unfortunately, this has led to a rapid decline in non-renewable P-rock reserves that may last only 50–100 years if mined at the current pace [1,3,4]. Heavy use of fertilizers not only threatens sustainable food production but also pollutes water resources, raising environmental concerns. To support sustainable agriculture and the environment, a widely accepted strategy is to minimize fertilizer use by breeding crops with higher P use efficiency (PUE). To achieve this goal, it is necessary to understand the molecular mechanism underlying Pi uptake from soil by roots, translocation from roots to the above-ground organs, storage into and remobilization from various subcellular compartments, and recycling from source (senescing leaves) to sink (young leaves, reproductive tissues and roots). All these processes require a specific set of transport proteins located in the plasma membrane of various plant cells and membranes of their subcellular compartments. 

In plants, phosphate transporters (PHTs) are classified into five major families, PHT1-5, based on their sequences and subcellular localizations [2,4]. The PHT1 family consists of H^+^/Pi symporters located in the plasma membrane of root cells for Pi uptake from the soil and translocation to the shoots [1,5]. It consists of nine members (AtPHT1;1–1;9) in *Arabidopsis*, among which AtPHT1;1 and AtPHT1;4 are the key players in Pi uptake [6,7]. AtPHT1;8 and AtPHT1;9 are also shown to function in Pi uptake and translocation from root to shoot under low-P [8,9]. AtPHT1;5 is involved in the redistribution of Pi from source to sink organs [10]. 

The other PHT family transporters are found in different subcellular compartments and play important roles in Pi storage and remobilization. AtPHT2;1 is the only PHT2 member in *Arabidopsis* that cotransports H^+^/Pi into chloroplasts [11,12]. The PHT3 family encodes three members in *Arabidopsis*, AtPHT3;1–3;3 (also referred as AtMPT1–3), that exchange Pi between the mitochondrial matrix and cytosol for ATP synthesis [13,14]. Overexpression of *AtPHT3;1/AtMPT3* in *Arabidopsis* resulted in a pleiotropic effect, suggesting a vital role of this gene in plant growth and development [14]. Among the six members in the AtPHT4 family, AtPHT4;1 is involved in Pi and pH homeostasis in chloroplasts, and plant defense against pathogens [15,16]. AtPHT4;2 mediates Na^+^-dependent Pi export from plastids in cells of sink organs [17]. AtPHT4;6 exports Pi from the *trans*-Golgi compartment to cytosol, essential for Golgi functions [18,19,20,21]. AtPHT5;1/AtVPT1 is located in the tonoplast to sequester excess Pi into vacuoles, and found to be essential for maintaining systemic Pi allocation for reproductive development along with AtPHT5;3/AtVPT3 [22,23,24].

With increasing effort in whole-genome sequencing, genome-wide identification of PHT families has been conducted in a number of plant species, including rice, maize, barley, potato, tomato, soybean, foxtail millet, sorghum, wheat, poplar, apple, and rapeseed [25,26,27,28,29,30,31,32,33,34,35,36,37]. Such genomic analysis of nutrient transporters has not been performed in *Camelina sativa* (false flax or gold-of-pleasure), an emerging oilseed crop for biofuel production. *Camelina* is an annual plant adapted to temperate growing regions, with centers of origin in southeastern Europe and southwestern Asia [38,39,40,41]. It belongs to the Crucifer family (Brassicaceae) that is closely related to *Arabidopsis thaliana*, and more distantly to *Brassica napus* [38]*. Camelina* has attracted considerable interest as an oil crop, primarily due to its numerous valuable agronomic traits that support sustainable agriculture. For example, a short life cycle of approximately 85–100 days, low input costs and ease of transformation using a simple floral dip procedure similar to that used for *Arabidopsis* make *Camelina* an attractive host for genetic and metabolic engineering [38,39,41,42]. In addition, *Camelina* has high tolerance to adverse environmental conditions (e.g., drought and low-nutrient status), resistance to pests and pathogens, and high oil content with a fatty acid profile suitable for fuel purposes [38,39,41,42,43]. Although *Camelina* has been shown to grow fairly well under P deficiency in field conditions [44,45], nothing is known on the genetic basis and gene networks enabling this plant to cope with low-P status. By utilizing the published genome sequence [38], we combine a comprehensive genomic and transcriptomic analyses of CsPHT transporters with low-P response of two *Camelina* accessions to initiate functional studies on the Pi transport system in this crop species.

## 2. Results

### 2.1. Identification and Phylogeny of the Five Major PHT Families

We identified 73 *CsPHT* genes in *Camelina* by blasting the coding sequences of known *Arabidopsis AtPHTs* against the *Camelina* genome. These genes belonged to the five PHT families, which include 33 *CsPHT1s*, 3 *CsPHT2s*, 9 *CsPHT3s*, 19 *CsPHT4s*, and 9 *CsPHT5s* (Supplementary Table S1). The proteins encoded by these *CsPHT* genes consisted of 309 to 704 amino acids, with the molecular weight (Mw) between 34.3 and 78.8 kDa, and isoelectric point (pI) value between 5.2 and 9.8. The number of transmembrane domains (TMDs) in CsPHT proteins varied between 4 and 13.

**Figure 1:**
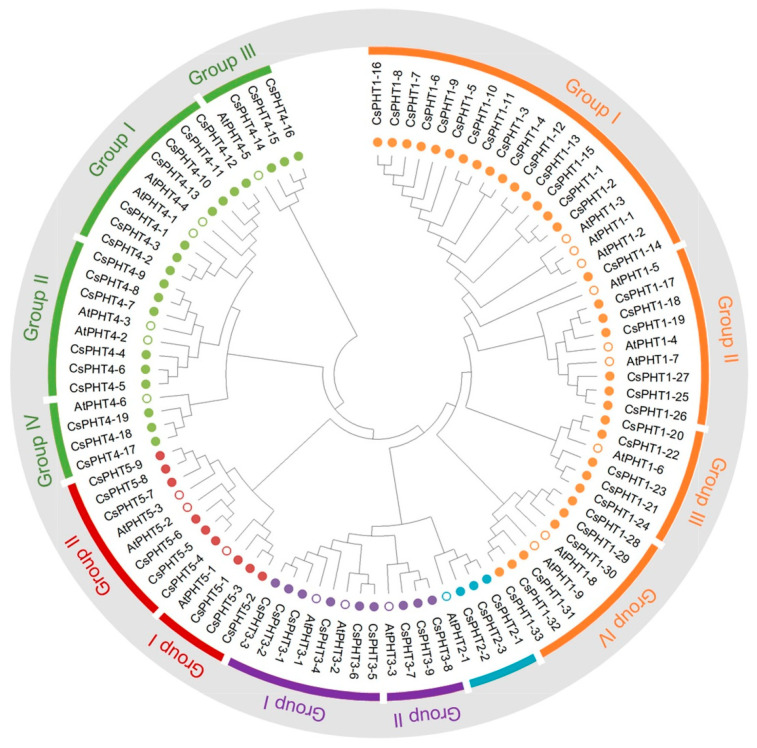
Evolutionary relationship between *Camelina* and *Arabidopsis* phosphate transporters (PHTs). The phylogenetic tree was constructed using neighbor-joining methods with the 1000 bootstraps in MEGA X program [46]. The PHTs of *Arabidopsis* are represented by open circles and *Camelina* by closed circles. Different PHT families are color-coded.

To investigate the evolutionary relationship between 73 *Camelina* and 22 *Arabidopsis* PHTs, their protein sequences were aligned to generate a phylogenetic tree using the neighbor-joining method in MEGA X [46] (Figure 1). Our results indicate that PHT1 family proteins were divided into four major groups, with AtPHT1;1/1;2/1;3 clustered in Group I along with 16 CsPHT1s. Groups II and IV each contained six CsPHT1s corresponding to their close homologous pairs, AtPHT1;4/1;7 and AtPHT1;8/1;9, respectively. While AtPHT1;5 was categorized into Group II, the *Camelina* genome appeared to lack homoeologs in this group. AtPHT1;6 along with five CsPHT1s were classified into Group III. The PHT2 family contained one AtPHT2;1 and three CsPHT2 members. Two distinct groups were found for the CsPHT3 family, with AtPHT3;1/3;2 and six CsPHT3 members in Group I, and AtPHT3;3 and three CsPHT3s in Group II. CsPHT4 family proteins formed four separate groups, with seven CsPHT4s corresponding to AtPHT4;1/4;4 homologous pairs in Group I, six CsPHT4s with AtPHT4;2/4;3 in Group II, and three CsPHT4s with AtPHT4;5 and AtPHT4;6 in Group III and IV, respectively. CsPHT5 family consisted of two distinct groups, AtPHT5;1 along with three CsPHT5s in Group I, and AtPHT5;2/5;3 with six CsPHT5s in Group II.

### 2.2. Chromosomal Distribution and Duplication of CsPHT Genes

**Figure 2:**
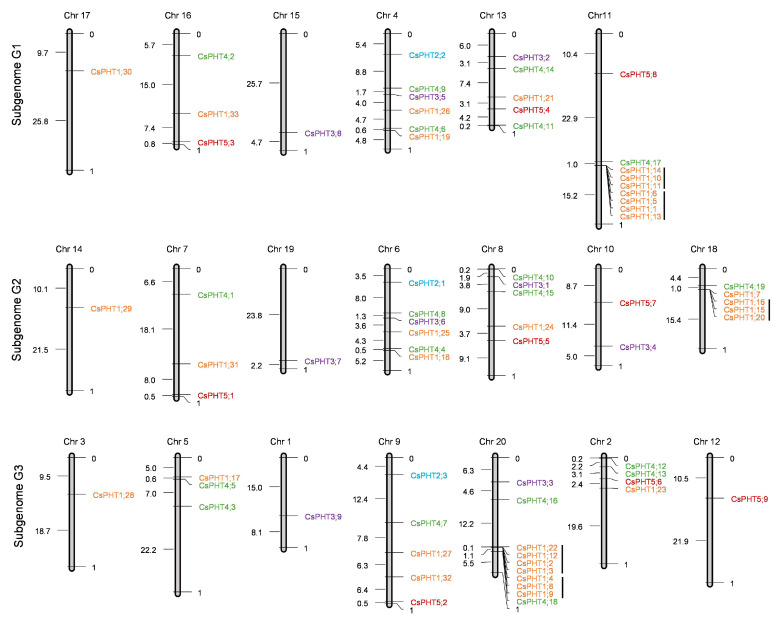
Chromosomal locations of *CsPHT* genes in the three sub-genomes. The genes were mapped using the MapDraw program [47]. Black lines next to the clustered genes represent tandem duplication events.

*CsPHT* genes were mapped to *Camelina* chromosomes based on their genomic locations using the MapDraw program [47]. Subgenome G1 and G3 each contained 25 *CsPHT* genes, and subgenome G2 harbored 23 *CsPHT* genes (Figure 2). This difference resulted from fewer *CsPHT1* genes in subgenome G2 as compared to subgenomes G1 and G3. Among the twenty chromosomes, *CsPHT1* genes were largely clustered on chromosomes 11, 18 and 20. *CsPHT2* and *CsPHT5* family genes were equally distributed across the three subgenomes. *CsPHT3* family genes were dispersed unevenly among the three subgenomes, with four *CsPHT3s* in subgenome G2, three in subgenome G1, and two in subgenome G3. While *CsPHT4* family genes were equally distributed between subgenome G1 and G2 with six genes each, one additional gene was found in subgenome G3.

*Camelina* has undergone whole-genome triplication event that retained triplet copies (homoeologs) for each *Arabidopsis* ortholog, with the majority of CsPHT families containing three homoeologous genes for each *AtPHT* gene [38] (Figure 1 and Supplementary Table S1). Among CsPHT families, the largest expansion beyond the ploidy norm was observed in CsPHT1 family. As a hexaploid, *Camelina* was expected to have 27 *CsPHT1* genes that correspond to nine *AtPHT* genes. However, we identified 33 *CsPHT1* genes, although no homoeologous genes were found for *AtPHT1;5*. The further expansion in the CsPHT1 family largely resulted from tandem duplication events (Figure 2 and Supplementary Figure S1). The presence of two or more homologous genes within the 200 kb region of the chromosome is considered tandem duplicated genes [26,36]. Close examination of *Camelina* genome revealed five tandem duplicated regions on chromosomes 11, 18 and 20 (Supplementary Figure S1).

### 2.3. Gene Structure and Protein Domain Analyses of CsPHT Members

**Figure 3:**
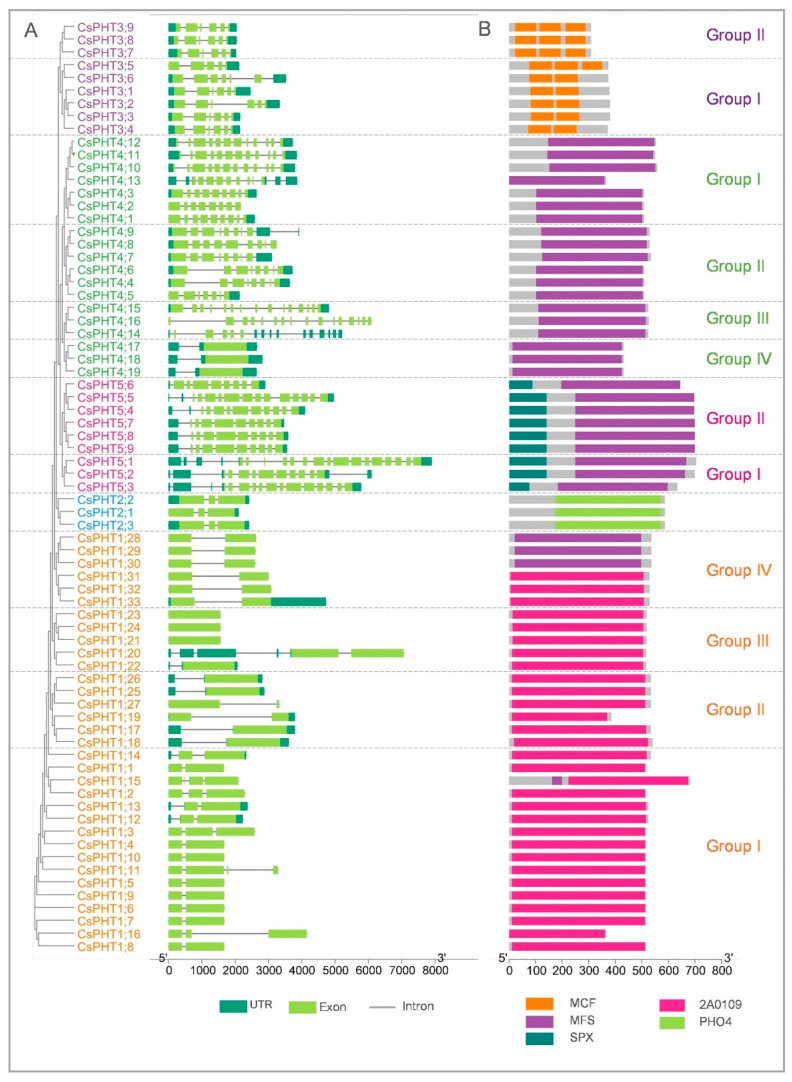
(**A**) Gene structures and (**B**) conserved protein domains of CsPHT members. The intron/exon structures were built using *Camelina* genome annotation file [38]. The protein domains were searched in NCBI-CDD database [50]. The graphics were generated using TBtools [48].

To inspect the structural diversity among CsPHT families, gene structures were built using the *Camelina* genome annotation file in TBtools [48]. Noticeably, the exon number and organization were more conserved within the same family, with one to two exons in the majority of *CsPHT1* genes, three exons in *CsPHT2s* and six exons in *CsPHT3s* (Figure 3A). However, the intron lengths within *CsPHT1/3* family genes varied between different groups, which might alter their encoding protein sequences and functions [49]. *CsPHT4* family genes showed the most diverse gene structures, with exons ranging broadly between 1 and 15. Among these, *CsPHT4* genes in Group IV shared highly conserved gene structures. *CsPHT5* family genes featured 9–16 exons, with Group II displaying a higher conservation in intron/exon organizations.

To find conserved domains within CsPHT family proteins, we scanned their protein sequences in NCBI’s Conserved Domain Database [50]. In general, some domains were specifically conserved within the same CsPHT family, while others were conserved across multiple CsPHT family proteins (Figure 3B). For instance, CsPHT1/4/5 family proteins all possessed conserved MFS (Major Facilitator Superfamily) or 2A0109 domains, which were found in Major Facilitator Superfamily proteins that transport Pi and other ions/substrates across plasma membrane and internal compartments including plastids, Golgi and vacuoles in *Arabidopsis* [16,19,21,22,23,51,52]. On the other hand, the PHO4 (PHOSPHATE4) domain was specifically conserved in CsPHT2 family proteins, similar to their *Arabidopsis* ortholog, AtPHT2;1, that transports Pi into chloroplasts [11,12]. CsPHT5 family proteins contained highly conserved SPX (SYG1/PHO81/XPR1)-MFS domains, which were specifically found on vacuolar Pi transporters in *Arabidopsis* and rice to maintain Pi homeostasis [22,23,52,53,54]. MCF (Mitochondrial Carrier Family) domains were uniquely conserved in CsPHT3 family proteins, implying their roles as Pi transporters in mitochondria [13,14,55]. However, Group I of the CsPHT3 family contained two MCF domains, while three were found in Group II, suggesting a possible functional divergence between these two groups in mitochondria.

### 2.4. Cis-Regulatory Elements of CsPHT Genes

**Figure 4:**
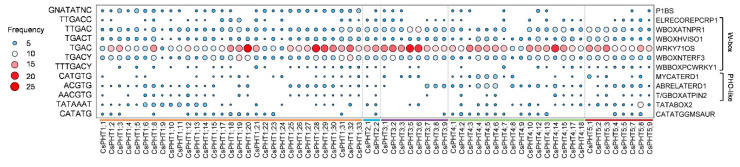
Frequency of Pi-starvation-related *cis*-elements in the 2-kb promoter region of *CsPHT* genes. The *cis*-elements were identified using the New PLACE database [56] and the dotplot was generated using TBtools [48].

Using 2-kb upstream regions of fully-sequenced *CsPHT* family genes (65/73) as queries, we searched the New PLACE database [56] and found 231 putative *cis*-regulatory elements (Supplementary Table S2). These *cis*-elements are known to govern gene expression in specific tissues (e.g., pollen, seed, root, flower), or in response to specific conditions, such as defense, phosphate, nitrogen, sugar, dehydration, energy, light, and other abiotic stresses [2,25,34]. Particularly relevant are those Pi starvation-related *cis*-elements, including P1BS, W-box, PHO-like, helix-loop-helix (HLH) and TATA-box-like elements (Figure 4). Among these, P1BS element was found in 40 of the 65 *CsPHT* genes examined. Most of the *CsPHT1* family genes (28/31) contained P1BS, suggesting these genes can be transcriptionally induced by PHR1 (PHOSPHATE RESPONSE1) transcriptional factor (TF) in response to low-P for efficient Pi uptake [57,58,59]. In addition, all *CsPHT1* genes contained a high frequency of several W-box elements, suggesting their expressions might be regulated by multiple WRKY TFs to modulate Pi uptake in response to the changing P status [60,61,62]. Similarly, the other *CsPHT* family genes might be transcriptionally regulated by WRKYs to mediate Pi distribution, as they also harbored a high frequency of those W-box elements. Several PHO-like, TATA-box-like (TATABOX2) and HLH (CATATGGMSAUR) elements were present in the majority of *CsPHT* family genes, except for *CsPHT3s*. These *cis*-elements were implicated to mediate low-P responsive expression, although not experimentally validated [2,63,64].

### 2.5. Expression Pattern of CsPHT Genes in Multiple Tissues

**Figure 5:**
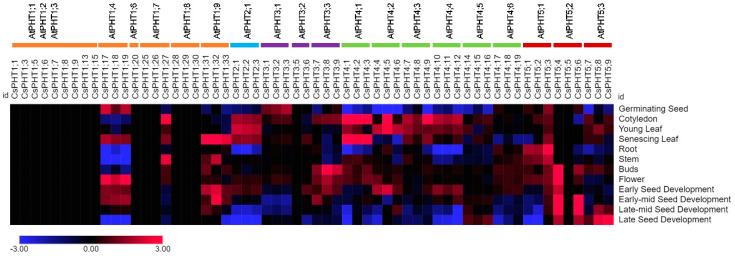
Expression pattern of *CsPHT* genes in 12 different tissues covering four developmental stages. The *Arabidopsis* PHT orthologs were shown on the top. The RNA-seq data (Log2 Ratio) were extracted from *Camelina* eFP browser (http://bar.utoronto.ca/efp_camelina/cgi-bin/efpWeb.cgi) [42], and the heatmap was generated using the MORPHEUS program (https://software.broadinstitute.org/morpheus/).

To investigate the expression pattern of *CsPHT* genes, we analyzed transcriptomic datasets generated for 12 different tissues covering four developmental stages [42]. The expression data for sixty out of seventy-three *CsPHT* family genes were analyzed (Figure 5). Because the datasets were acquired under normal growth conditions, the majority of *CsPHT1* family genes were not expressed, consistent with the presence of low-P responsive *cis*-elements in the promoter sequences of these genes. Nevertheless, we found both conservation and divergence in the expression patterns among the three close homoeologous genes for each *Arabidopsis* ortholog. For instance, *CsPHT1;7*–*1;19*, the three homoeologs for *AtPHT1;4,* displayed similar expression patterns in germinating seeds, senescing leaves, flowers, and seed development. In contrast, *CsPHT1;31*–*1;33*, homoeologs for *AtPHT1;9,* were expressed similarly in senescing leaves, but differed sharply in stems, flower buds and seeds. In addition, *CsPHT1;27* corresponding to *AtPHT1;7* displayed a dominant expression during the early and vegetative stages, while no expression was detected for the other two close homoeologs.

The three *CsPHT2* genes were exclusively expressed in leaves during the early and vegetative stages. The closely-related *CsPHT3;1*–*3;3* genes of *AtPHT3;1* shared tissue-specific expression in germinating seeds, whereas *CsPHT3;7*–*3;9*, homoeologs for *AtPHT3;3*, were more broadly expressed in cotyledons and reproductive organs. Most of the *CsPHT4* family genes, including *CsPHT4;1–4;12*, were expressed in leaves, but only *CsPHT4;1*–*4;3,* close homoeologs for *AtPHT4;1,* showed preferential expression in senescing leaves. *CsPHT4;1,* –*4;3,* –*4;4* and –*4;6* were additionally expressed at low level in flowers, and *CsPHT4;4*–*4;6* and *4;9*–*4;12* in early seed development. Division in tissue expressions was observed in *CsPHT5* family genes. For example, only *CsPHT5;1*–*5;3*, close homoeologs for *AtPHT5;1/AtVPT1,* were expressed in roots and stems during vegetative growth, with *CsPHT5;3* showing a ubiquitous and dominant expression. On the other hand, the rest of the *CsPHT5* genes except for *CsPHT5;5* and –*5;7* were expressed during the later stages of the seed development, with *CsPHT5;4* exhibiting a broader expression.

### 2.6. Camelina’s Phenotype and Expression Analysis of CsPHT Genes in Response to Low-P

**Figure 6:**
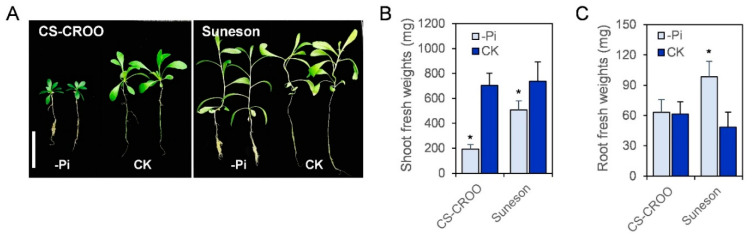
(**A**) Phenotype, (**B**) shoot biomass, and (**C**) root biomass of the low-P-sensitive CS-CROO and the low-P-tolerant Suneson grown under low-P (-Pi, 10 µM) and the control (CK, 312 µM) conditions for four weeks. White scale bar represents 5 cm. The biomass data represents the mean ± SD of one of the three experiments (*n* = 10). Asterisk indicates a significant difference (* *p* < 0.0005) between -Pi and CK conditions.

To examine low-P response in *Camelina*, we grew two different accessions under low-P (-Pi) and control (CK) conditions (Figure 6). Phenotypically, Suneson displayed a high tolerance to low-P, whereas CS-CROO was highly sensitive (Figure 6A). Suneson retained ~70% of shoot biomass under low-P relative to the control condition, whereas CS-CROO only maintained ~30% (Figure 6B). In addition, Suneson accumulated a significantly higher root biomass under low-P compared to the control, whereas no difference was found for CS-CROO (Figure 6C).

**Figure 7:**
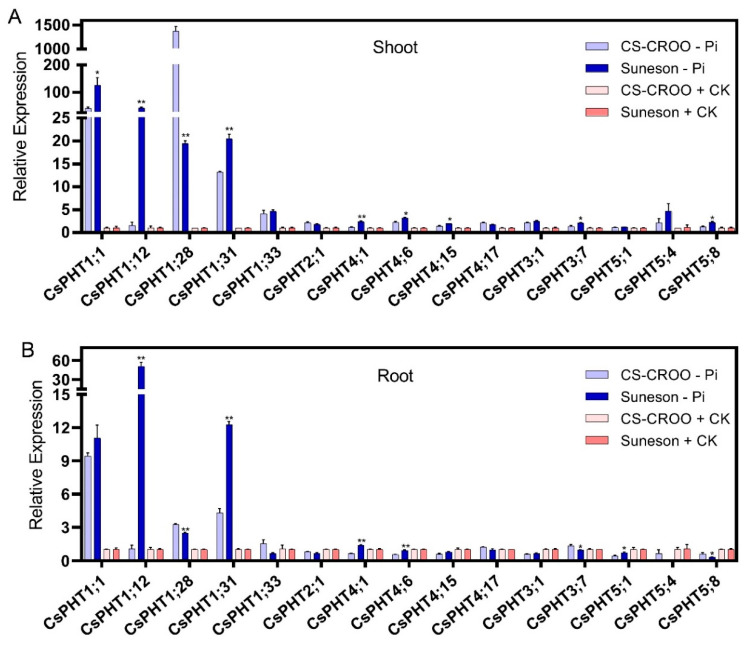
Expression analysis of 15 selected *CsPHT* genes in (**A**) Shoot and (**B**) Root of the low-P-sensitive CS-CROO and the low-P-tolerant Suneson treated under low-P (-Pi, 10 µM) and the control (CK, 312 µM) conditions for four weeks, as revealed by qRT-PCR. The mean ± SD of three biological replicates are presented. Asterisks indicate a significant difference (* *p* < 0.01 and ** *p* < 0.001) in gene expression between two *Camelina* accessions after Pi treatments.

To understand the genetic basis underlying low-P response of the two *Camelina* accessions, we analyzed the expression patterns of 15 genes from the five CsPHT families under low-P (-Pi) and the control (CK) conditions using quantitative real-time PCR (Figure 7). The high sequence homology between the closely-related genes and their homoeologous/paralogous genes posed a challenge in designing gene-specific primers, thus the expression patterns of only a subset of *CsPHT* genes were analyzed. In response to low-P, the majority of *CsPHT1* genes analyzed were highly upregulated in both shoots and roots of *Camelina* (Figure 7A,B). Interestingly, a number of these genes were expressed at a higher level in the low-P-tolerant Suneson as compared to the low-P-sensitive CS-CROO, including *CsPHT1;1*, –*1;12* and –*1;31* in shoots, and *CsPHT1;12* and –*1;31* in roots. In addition, several *CsPHT4* family genes were upregulated at a slightly higher level in Suneson than CS-CROO under low-P, including *CsPHT4;1*, –*4;6* and –*4;15* in shoots, and *CsPHT4;1* and –*4;6* in roots. Suneson also exhibited slightly higher expression of *CsPHT3;7* and –*5;8* in shoots, and *CsPHT5;1* in roots compared to CS-CROO under low-P. These genes might contribute to the better Pi uptake and distribution capabilities of Suneson in response to low-P.

## 3. Discussion

### 3.1. Expansion and Structural Conservation in CsPHT Families

*Camelina* is an oilseed crop that is capable of growing on marginal lands with modest input of fertilizers (and other resources), thus serving as a strong candidate crop that may utilize non-agriculture lands for fuel purposes [38,40,42,43]. However, *Camelina*’s response to low-nutrient status has not been studied at the genome level. In this study, we identified 73 genes encoding CsPHT transporters in the *Camelina* genome and analyzed their expression patterns in various tissues and in response to low-P, providing a road map to understand the transport and utilization of Pi in this promising oil crop.

The vast expansion of CsPHT families in *Camelina* mainly resulted from the whole-genome triplication event that occurred 17 million years ago [38]. In addition, gene duplications further expanded these CsPHT families. In particular, these events drove major evolutionary changes in the composition of the CsPHT1 family. Although there is an overall expansion of genes beyond the ploidy norm (33 vs. 27), a specific loss of all homoeologs for *AtPHT1;5* was identified, presenting an interesting point for functional analysis in the future. Tandem gene duplication events were the major driving force for the expansion of these *CsPHT1* genes on chromosome 11, 18 and 20. These tandem duplicated genes largely correspond to *AtPHT1;1/1;2/1;3* orthologs, which are functionally redundant genes that function as key players in Pi uptake [7,65]. The significant expansion of this gene family in *Camelina* might contribute to the robust Pi acquisition and low-P tolerance in this polyploidy plant.

The three CsPHT2 members might share similar functions to their ortholog, AtPHT2;1 as H^+^/Pi symporters since their gene structures, protein sizes and PHO4 domain were highly conserved [11,12]. *Camelina* has nine CsPHT3 members with MCF domains, which are also found in AtPHT3 transporters that affect mitochondrial respiration processes [13,14,55]. However, CsPHT3s in Group I differ in exon/intron organizations with two MCF domains, while those in Group II contain highly conserved gene structures with three MCF domains. Transport activity analysis of these two diverging groups might provide clues to the significance of this domain for Pi transport mechanism in mitochondria. 

*Camelina* has 19 CsPHT4 members clustered largely in Group I and II with diverse gene structures, although all display conserved MFS domains. This suggests that CsPHT4 members might have a wide range of functions, as observed in *Arabidopsis* that transport Pi and other solutes, such as ascorbate, in different subcellular compartments, and are involved in plant defense and salt tolerance [15,16,19,20,21]. Nine CsPHT5 members are found in *Camelina* with conserved SPX-MFS domains that are also found on vacuolar Pi-transporters in *Arabidopsis* and rice that control vacuolar Pi fluxes [22,23,52,53,54]. For instance, Pi influx is mediated by *Arabidopsis* AtPHT5;1/AtVPT1, and rice OsSPX-MFS1 and OsSPX-MFS3, whereas Pi efflux from vacuoles is mediated by VPEs in rice and *Arabidopsis* [22,23,52,53]. In addition, these SPX-MFS proteins might have Pi-sensing abilities [66], which is worth further investigation.

### 3.2. CsPHT Gene Expressions in Tissues and in Response to Low-P

Understanding when and where a gene is expressed could provide valuable information about its role in plant growth and development. Therefore, we analyzed the expression pattern of *CsPHT* genes in various plant tissues of *Camelina* DH55 genotype [42]. In general, the *CsPHT1* genes displayed a broad expression in multiple tissues, implying their roles in Pi transport across various tissues throughout plant growth and development. However, these genes were not expressed in roots under the normal growth condition. This might be because a large number of CsPHT1 transporters are encoded in *Camelina*, and therefore, a basal expression level in roots might be adequate to efficiently uptake Pi under sufficient-P condition. Nevertheless, these *CsPHT1* genes were transcriptionally induced under low-P, probably because these genes harbor P1BS (PHR1 binding site) and several W-box (WRKY binding site) elements on their promoter regions. AtPHR1 and AtWRKY45/75 in *Arabidopsis*, and OsPHR2 and OsWRKY74 in rice are shown to bind to those *cis*-elements to promote efficient Pi acquisition and translocation especially under low-P stress [57,58,59,60,61,67,68,69]. These Pi transport processes are mediated by *AtPHT1;1*, *–1;4*, *–1;8* and *–1;9* in *Arabidopsis*, and *OsPHT1;2*, *–1;3*, *–1;9* and *–1;10* in rice [6,7,8,9,70,71,72].

The *CsPHT2* genes displayed tissue-specific expression in leaves, similar to *Arabidopsis* and rice, suggesting their conserved roles in Pi-transport into chloroplasts [11,12,73,74]. *CsPHT3;7–3;9* and their ortholog, *AtPHT3;3* showed similar expression patterns in floral organs [75], indicating their importance in reproductive development. However, *CsPHT3;1–3;3* genes exhibited tissue-specific expression in germinating seeds, in contrast to their orthologous gene, *AtPHT3;1*, that showed ubiquitous expression [75]. This might infer a neofunctionalization of these *CsPHT3* genes in seed germination. The PHT3 family is the least studied among the five PHT families in *Arabidopsis* due to the lethality of homozygous mutants [14]. Thus, it would be interesting to decipher their physiological roles in *Camelina* that contains multiple copies, and knocking out one or more homoeologous genes might not pose such a challenge. 

Many *CsPHT4* genes were highly expressed in leaves, similar to their orthologous genes in *Arabidopsis*, suggesting their putative roles in Pi transport into chloroplasts, as observed for AtPHT4;1 [16,18,76]. However, the dominant expression of *CsPHT4;1–4;3* in senescing leaves confers their additional roles in source to sink Pi transport, as observed for AtPHT4;6 [21]. Intriguingly, the majority of *CsPHT5* genes were expressed in flowers and seeds, and the significance of these vacuolar transporters in maintaining Pi homeostasis during reproductive development has recently been confirmed in *Arabidopsis* [24]. Among the *CsPHT5* family genes, *CsPHT5;3* showed a dominant expression from early to vegetative growth, whereas *CsPHT5;4* was dominant from flower to seed development. Thus, the functional studies of these two genes as key vacuolar Pi-transporters are worth further investigation. 

To understand *Camelina*’s response to low-P at a genetic level, we compared the growth and gene expression of two *Camelina* accessions under low-P and the control conditions. Our results indicate Suneson as the low-P-tolerant genotype, and CS-CROO as the low-P-sensitive genotype. Suneson might have a better Pi utilization compared to CS-CROO based on the gene expression analysis. For example, the high expression of *CsPHT1;12* and *–1;31* genes in roots of Suneson under low-P suggests a better Pi-uptake capability than CS-CROO, as demonstrated by the roles of these *PHT1* genes in *Arabidopsis* [7,8]. The other *CsPHT* family genes are likely involved in Pi distribution involving Pi remobilization from subcellular compartments and Pi recycling from source to sink in order to maintain cellular Pi homeostasis. For example, the higher expression of *CsPHT4;6* in shoots and roots of Suneson implies a better Pi remobilization, as illustrated by the function of its *Arabidopsis* ortholog, *AtPHT4;2* in exporting Pi from plastids [17] In addition, *CsPHT4;1* was expressed at a higher level in shoots of Suneson than CS-CROO, and its tissue-specific expression in senescing leaves infers the potential role of this gene in recycling Pi from older to younger leaves under low-P. Similarly, although *CsPHT1;1*, *–1;12* and *–1;31* genes are putative Pi-uptake genes, their higher expressions in shoots of Suneson suggest their additional functions in distributing Pi in leaves, which remain unexplored. In *Arabidopsis*, AtPHT1;5 was shown to be involved in this process [10], although no homoeologs corresponding to this gene were found in *Camelina*. Thus, other CsPHT1 members along with CsPHT4s might compensate for the loss. Moreover, the higher expression of *CsPHT5;8* in shoots, and *CsPHT5;1* in roots of Suneson might maintain vacuolar Pi homeostasis better than CS-CROO under low-P [22,23,24]. Additionally, the higher expression of *CsPHT3;7* in shoots of Suneson might be important for respiration processes in mitochondria, as observed in *Arabidopsis* [13,14,55].

Further expression analysis is required as we only covered a subset (15/73) of *CsPHT* genes for which we could design gene-specific primers. Global transcriptomic analysis of low-P-tolerant and -sensitive genotypes coupled with GWAS analysis of large *Camelina* varieties under low-P will aid in dissection of the gene-networks involving PHTs, TFs and signaling components that contribute to the higher PUE in *Camelina* such as Suneson. In addition, this global transcript analysis will provide clues regarding the functional dominance of one homoeolog over the others in response to low-P stress. Further genetic, molecular, biochemical and electrophysiological analyses of the candidate CsPHT transporters will elucidate the biological significance and function of these transporters in Pi acquisition and distribution. These approaches are critical for future breeding efforts to engineer crops with high PUE for sustainable agriculture.

## 4. Materials and Methods

### 4.1. Genome-Wide Identification of CsPHT Families in Camelina

To identify the candidate CsPHT members, the coding sequences of all known AtPHT members of *Arabidopsis thaliana* were individually blasted against the *Camelina sativa* database in the National Center for Biotechnology Information (NCBI, https://blast.ncbi.nlm.nih.gov/). The coding sequences of AtPHT members were obtained from The *Arabidopsis* Information Resource (TAIR10) database (https://www.arabidopsis.org/) [77]. The choice of candidate PHTs was based on the E-value over 1 × 10^−10^ and query over 60%. Various splicing variants of a gene and incomplete genes with short length were discarded. The CsPHT chromosomal locations and their genomic DNA were obtained from NCBI. The coding sequences and deduced protein sequences were retrieved from the Kyoto Encyclopedia of Genes and Genomes/KEGG Genome: *Camelina sativa* (https://www.genome.jp/). The Locus IDs were obtained from “BLAST Database” in The Prairie Gold Novel Industrial Oilseed Crops for Canada Database (http://www.camelinadb.ca/). The properties of putative PHT proteins such as number of amino acids, molecular weights (Mw), and isoelectric point (pI) were calculated using ProtParam (http://web.expasy.org/protparam/). Transmembrane domains (TMDs) were predicted using TMpred (https://embnet.vital-it.ch/software/TMPRED_form.html), with a score above 500 considered as significant.

### 4.2. Phylogenetic Analysis and Chromosome Mapping

Multiple amino acid alignment of the CsPHT proteins of *Camelina* and *Arabidopsis* was generated using ClustalW. The *Arabidopsis* AtPHT protein sequences were obtained from TAIR10 database (https://www.arabidopsis.org/) [77]. The phylogenetic tree was constructed using the neighbor-joining method with the 1000 bootstrap in MEGA version X program [46]. The chromosomal locations of *CsPHT* genes were mapped using the MapDraw program [47]. Tandem duplicated genes were identified in *EnsemblPlants* (http://plants.ensembl.org/Camelina_sativa/Info/Index) by close examination of the chromosomal regions of all clustered genes found on the three subgenomes (Supplementary Figure S1). Homologous genes in close proximity (<200 kb) were considered tandem duplicated genes [26,36].

### 4.3. Gene Structure and Protein Domain Analyses

The gene structures were generated using *Camelina* genome annotation GFF file (downloaded from http://www.camelinadb.ca/) [38]. The conserved protein domains were identified by scanning the protein sequences in the NCBI Conserved Domain Database (NCBI-CDD) search (https://www.ncbi.nlm.nih.gov/Structure/bwrpsb/bwrpsb.cgi) [50]. The graphics of both gene structures and conserved protein domains were created using TBtools [48].

### 4.4. Cis-Regulatory Element Identification

Promoter sequences located 2-kb upstream of the transcription start sites (ATG codons) of *CsPHT* genes were downloaded from NCBI (https://blast.ncbi.nlm.nih.gov/). The multiple promoter sequences of *CsPHT* genes were scanned in New PLACE (https://www.dna.affrc.go.jp/PLACE/?action=newplace) [56] to identify the abundance of 231 *cis*-regulatory elements (Supplementary Table S2). The subset of data representing Pi-starvation-related *cis* elements was plotted using TBtools [48].

### 4.5. In-Silico Expression Analysis of CsPHT Genes

The RNA-Seq data (Log2 Ratio) of *CsPHT* genes across 12 different tissues covering 4 developmental stages of the *Camelina* life cycle were extracted from *Camelina* eFP browser (http://bar.utoronto.ca/efp_camelina/cgi-bin/efpWeb.cgi) [42]. The developmental stages included early growth (germinating seed and cotyledon), vegetative growth (young leaf, root, stem and senescing leaf), flower development (buds and flower) and seed development (early, early-mid, late-mid and late). The heatmap of gene expressions was generated using the MORPHEUS program (https://software.broadinstitute.org/morpheus/).

### 4.6. Plant Materials and Growth Conditions

The sources of seeds for the two *Camelina* accessions, CS-CROO and Suneson, are listed in Supplementary Table S3. The seeds were sterilized in 20% bleach, and washed three times with water. The sterilized seeds were stratified in water at 4 °C for 15 days before plating on the medium. Seeds were germinated on ¼ Murashige and Skoog (MS) Medium (Ref: MSP09-100LT, Caisson Labs, Smithfield, UT, USA) with 1% sucrose, 0.8% phytoblend for 5 days, then seedlings were transferred to hydroponic solution containing ¼ MS for 9 days. The two-week-old plants were divided into 2 groups, and grown in low-P (-Pi, 10 µM) and the control (CK, 312 µM) hydroponic culture for 4 weeks. The plants were grown in a greenhouse with 12 h/12 h of light/dark cycle at a temperature between 18 and 22 °C. Shoot and root tissues were then separately collected to measure fresh weights. The experiment was repeated three times, with each experiment composed of 5–10 plants per accession for each treatment. The mean and standard deviation (SD) of one representative experiment containing 10 plants per accession for each treatment is presented. Student’s *t* test was used to compare biomass between -Pi and CK conditions for each accession.

### 4.7. RNA Isolation and cDNA Synthesis

Shoot and root tissues were separated, and quickly frozen in liquid nitrogen. Total RNA was extracted using TRIzol Reagent (Invitrogen, Carlsbad, CA, USA). After treatment with DNase I (New England Biolabs, Ipswich, MA, USA), 2 μg of total RNA was subjected to reverse transcription reaction using the M-MLV Reverse Transcriptase (Promega, San Luis Obispo, CA, USA) at 42 °C for 1 h. The resulting cDNA was used for PCR amplification with the gene-specific primers. qRT-PCR analysis was performed using the CFX96^TM^ Real-Time System (Bio-Rad, Hercules, CA, USA). Each PCR mixture contained 10 μL SYBR Green Master Mix (Bio-Rad, Hercules, CA, USA), a pair of 0.5 μM gene-specific primers, and 5 μL of the cDNA sample (∼100 ng) in a final volume of 20 μL. Alpha tubulin (*CsTUA,* accession XM_010481098.1) was used as the internal control [78]. The specific primer sequences for the control and *CsPHT* genes are listed in Supplementary Table S4. Primers were designed using NCBI primer-BLAST [79] with a desired product size >200 bp, and at least 5 total mismatches to unintended targets. Due to the high sequence homology among the close homoeologs/paralogs, the expression analysis was performed on only a subset of genes that show high primer specificity, confirmed by the melting curve analysis. Genes with one single peak in the melting curve analysis were included in the data. The relative expression level of each gene was calculated based on the comparative threshold cycle method using the internal control, and was further normalized to the control expression values measured for the CK condition. The experiment was repeated three times with three technical replicates, and the values represent the mean and SD. Expression difference in *CsPHTs* between the two *Camelina* accessions after Pi treatments was assessed by Student’s *t* test (* *p* < 0.01 and ** *p* < 0.001).

## 5. Conclusions

We identified and characterized 73 *CsPHT* genes in *Camelina* that share more similar gene structures and protein domains within the same CsPHT family. Polyploidization and tandem duplication events drove the vast expansion of these CsPHT families in *Camelina*. *Cis*-element P1BS was more specifically conserved in the promoter regions of *CsPHT1* genes to mediate Pi uptake under low-P. In silico RNA-seq analysis suggested the importance of *CsPHT1* genes throughout plant development, *CsPHT2s* and *CsPHT4s* in vegetative growth, *CsPHT5s* in reproductive development, and *CsPHT3s* in early growth and reproductive stages. However, several genes showed dominant expression compared to their close homoeologous genes including *CsPHT1;27*, *–5;3* and *–5;4*. A popular Camelina variety, Suneson showed better tolerance to low-P than CS-CROO, with higher expression of *CsPHT1;12*, *–1;31*, *–4;1*, *–4;6* and *–5;1* in roots, and *CsPHT1;1*, *–1;12*, *–1;31*, *–3;7*, *–4;1*, *–4;6*, *–4;15* and *–5;8* in shoots. More in-depth analysis at global transcript level and/or GWAS of a large pool of *Camelina* accessions under low-P could provide a more holistic view of specific Pi-transporters and regulators that are involved in providing enhanced low-P tolerance to *Camelina* accessions (e.g., Suneson). This could serve as a better framework for future functional studies of CsPHT transporters in *Camelina*, and as a model for other crop species.

## Supplementary Materials

Supplementary Materials can be found at https://www.mdpi.com/1422-0067/21/21/8365/s1.
