# Supplementary material for: Genome-Wide Analysis of the Five Phosphate Transporter Families in Camelina sativa and Their Expressions in Response to Low-P
Source: Int J Mol Sci. 2020 Nov 7;21(21):8365. doi: 10.3390/ijms21218365 (PMC7664626; doi:10.3390/ijms21218365)
Supplement: Supplementary file 1 [file ijms-21-08365-s001.zip › Supplementary Materials.docx]

Supplementary Materials

Genome-Wide Analysis of the Five Phosphate Transporter Families in *Camelina sativa* and Their Expressions in Response to Low-P

**
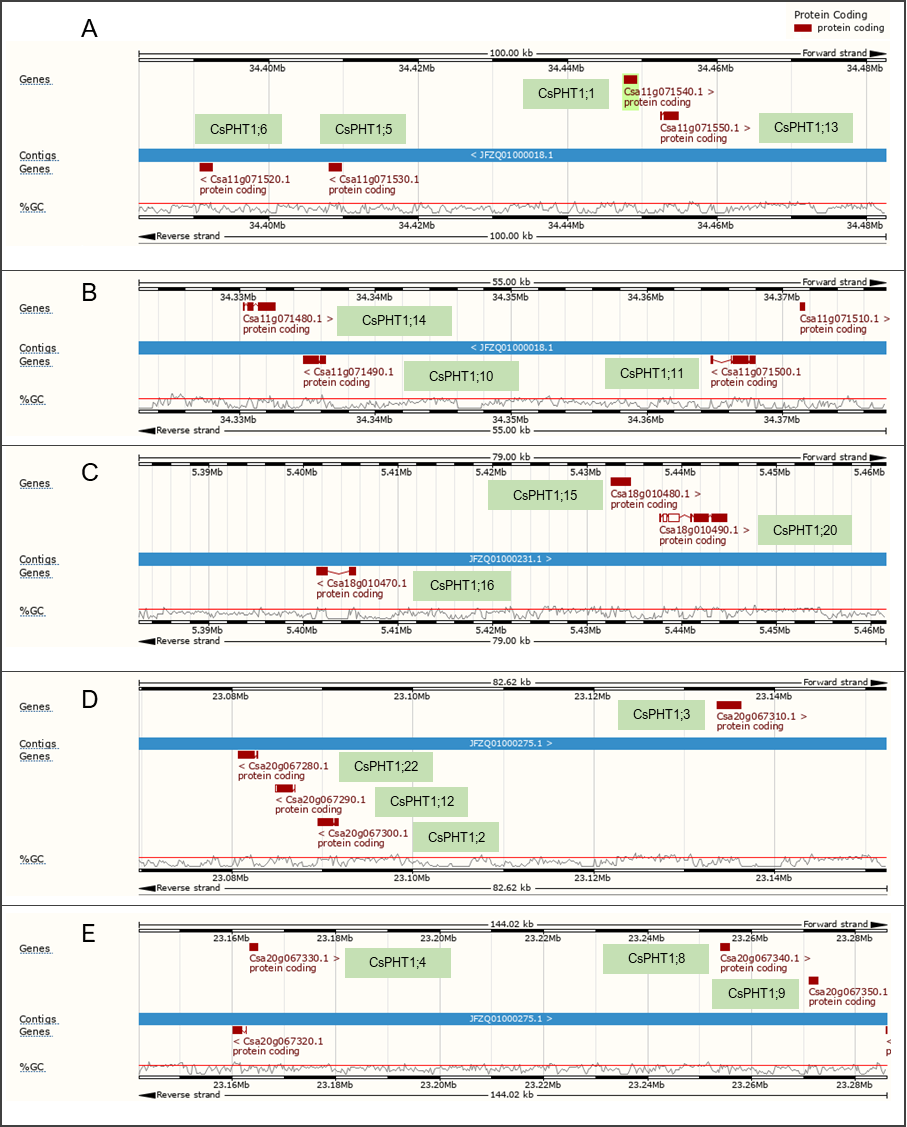
**

**Figure S1.** (**A**–**E**) Five tandem duplicated regions of *CsPHT1* family genes. The gene regions within the *Camelina* genome were visualized in *EnsemblPlants* (http://plants.ensembl.org/Camelina_sativa/Info/Index).

**Table S1.** The properties of 73 phosphate transporter (CsPHT) members identified in *Camelina sativa.*

| ***A. thaliana* Gene** | ***C. sativa* Gene** | **Locus ID** | **NCBI Gene ID** | **Genome Location** | **Gene Length (bp)** | **Protein Size** | **Mw (kDa)** | **pI** | **TMD** |
| --- | --- | --- | --- | --- | --- | --- | --- | --- | --- |
| *AtPHT1;1*  *AtPHT1;2*  *AtPHT1;3* | *CsPHT1;1* | Csa11g071540.1 | 104725125 | Chr11: 34447559–34449226 | 1554 | 517 | 56.9 | 8.9 | 11 |
|  | *CsPHT1;2* | Csa20g067300.1 | 104771484 | Chr20: 23089482–23091145 | 1554 | 517 | 56.9 | 8.9 | 11 |
|  | *CsPHT1;3* | Csa20g067310.1 | 104771485 | Chr20: 23133274–23136312 | 1554 | 517 | 56.8 | 8.9 | 11 |
|  | *CsPHT1;4* | Csa20g067330.1 | 104771486 | Chr20: 23163050–23165166 | 1554 | 517 | 56.8 | 8.9 | 11 |
|  | *CsPHT1;5* | Csa11g071530.1 | 104725122 | Chr11: 4407992–34409716 | 1554 | 517 | 56.8 | 8.9 | 11 |
|  | *CsPHT1;6* | Csa11g071520.1 | 104725124 | Chr11: 34390594–34392799 | 1554 | 517 | 56.8 | 8.9 | 11 |
|  | *CsPHT1;7* | Csa18g010450.1 | 104760616 | Chr18: 5356497–5358446 | 1554 | 517 | 56.8 | 8.9 | 11 |
|  | *CsPHT1;8* | Csa20g067340.1 | 104771487 | Chr20: 23253748–23255987 | 1554 | 517 | 56.8 | 8.9 | 11 |
|  | *CsPHT1;9* | Csa20g067350.1 | 104771488 | Chr20: 23270739–23272750 | 1554 | 517 | 56.8 | 8.9 | 11 |
|  | *CsPHT1;10* | Csa11g071490.1 | 104725120 | Chr11: 34334575–34336343 | 1554 | 517 | 56.8 | 8.9 | 11 |
|  | *CsPHT1;11* | Csa11g071500.1 | 104725121 | Chr11: 34366281–34367975 | 1554 | 517 | 56.8 | 8.9 | 11 |
|  | *CsPHT1;12* | Csa20g067290.1 | 104771483 | Chr20: 23084773–23087017 | 1575 | 524 | 57.7 | 8.6 | 12 |
|  | *CsPHT1;13* | Csa11g071550.1 | 104725126 | Chr11: 34452457–34454809 | 1575 | 524 | 57.8 | 8.8 | 12 |
|  | *CsPHT1;14* | Csa11g071480.1 | 104725119 | Chr11: 34330271–34332704 | 1608 | 535 | 58.4 | 8.8 | 12 |
|  | *CsPHT1;15* | Csa18g010480.1 | 104763156 | Chr18: 5428765–5434620 | 2043 | 680 | 75.2 | 9.2 | 13 |
|  | *CsPHT1;16* | Csa18g010470.1 | 109130617 | Chr18: 5401307–5402565 | 1101 | 366 | 40.6 | 8.3 | 7 |
| *AtPHT1;4* | *CsPHT1;17* | Csa05g014350.1 | 104785556 | Chr5: 5014985–5018549 | 1605 | 534 | 58.7 | 8.4 | 11 |
|  | *CsPHT1;18* | Csa06g042880.1 | 104792707 | Chr6: 21171205–21174625 | 1626 | 541 | 59.6 | 8.7 | 11 |
|  | *CsPHT1;19* | Csa04g052890.1 | 104784270 | Chr4: 25092929–25096715 | 1161 | 386 | 42.0 | 6.6 | 9 |
| *AtPHT1;6* | *CsPHT1;20* | Csa18g010490.1 | 104760617 | Chr18: 5442928–5444835 | 1554 | 517 | 56.5 | 9.0 | 12 |
|  | *CsPHT1;21* | Csa13g041920.1 | 104736909 | Chr13: 16476804–16478360 | 1557 | 518 | 56.4 | 8.8 | 11 |
|  | *CsPHT1;22* | Csa20g067280.1 | 104771482 | Chr20: 23080792–23082821 | 1554 | 517 | 56.4 | 9.1 | 12 |
|  | *CsPHT1;23* | Csa02g023260.1 | 104749571 | Chr2: 7988533–7990089 | 1557 | 518 | 56.2 | 8.8 | 11 |
|  | *CsPHT1;24* | Csa08g034850.1 | 104709547 | Chr8: 14895358–14896914 | 1557 | 518 | 56.4 | 8.8 | 11 |
| *AtPHT1;7* | *CsPHT1;25* | Csa06g029160.1 | 104791742 | Chr6: 16352685–16355511 | 1608 | 535 | 58.4 | 8.7 | 11 |
|  | *CsPHT1;26* | Csa04g040820.1 | 104781337 | Chr4: 19796700–19799466 | 1608 | 535 | 58.4 | 8.7 | 11 |
|  | *CsPHT1;27* | Csa09g064460.1 | 104712074 | Chr9: 24546604–24549170 | 1608 | 535 | 58.4 | 8.7 | 11 |
| *AtPHT1;8* | *CsPHT1;28* | Csa03g024720.1 | 104776208 | Chr3: 9470208–9473149 | 1611 | 536 | 59.1 | 6.5 | 12 |
|  | *CsPHT1;29* | Csa14g026060.1 | 104740779 | Chr14: 10079166–10082116 | 1611 | 536 | 59.1 | 6.5 | 12 |
|  | *CsPHT1;30* | Csa17g026750.1 | 104756440 | Chr17: 9722033–9725135 | 1611 | 536 | 59.2 | 6.5 | 12 |
| *AtPHT1;9* | *CsPHT1;31* | Csa07g047990.1 | 104702405 | Chr7: 24682226–24685501 | 1590 | 529 | 58.4 | 8.9 | 12 |
|  | *CsPHT1;32* | Csa09g081170.1 | 104713308 | Chr9: 30797367–30800766 | 1593 | 530 | 58.5 | 8.5 | 12 |
|  | *CsPHT1;33* | Csa16g040570.1 | 104751540 | Chr16: 20731687–20736691 | 1593 | 530 | 58.5 | 8.5 | 12 |
| *AtPHT2;1* | *CsPHT2;1* | Csa06g006270.1 | 104790285 | Chr6: 3480294–3482820 | 1764 | 587 | 61.2 | 9.3 | 12 |
|  | *CsPHT2;2* | Csa04g012130.1 | 104779868 | Chr4: 5383632–5386127 | 1764 | 587 | 61.3 | 9.4 | 12 |
|  | *CsPHT2;3* | Csa09g010040.1 | 104710447 | Chr9: 4351594–4354039 | 1764 | 587 | 61.2 | 9.3 | 12 |

**Table S1.** *Cont.*

| ***A. thaliana Gene*** | ***C. sativa Gene*** | **Locus ID** | **NCBI Gene ID** | **Genome Location** | **Gene Length (bp)** | **Protein Size** | **Mw (kDa)** | **pI** | **TMD** |
| --- | --- | --- | --- | --- | --- | --- | --- | --- | --- |
| *AtPHT3;1/*  *AtMPT3* | *CsPHT3;1* | Csa08g006050.1 | 109124421 | Chr8: 2089525–2091846 | 1140 | 379 | 40.3 | 9.3 | 4 |
|  | *CsPHT3;2* | Csa13g016810.1 | 104705671 | Chr13: 5989032–5992238 | 1146 | 381 | 40.5 | 9.3 | 4 |
|  | *CsPHT3;3* | Csa20g020500.1 | 104769633 | Chr20: 6286253–6288550 | 1146 | 381 | 40.5 | 9.3 | 4 |
|  | *CsPHT3;4* | Csa10g042780.1 | 104719447 | Chr10: 20121608–20123709 | 1119 | 372 | 39.7 | 9.3 | 4 |
| *AtPHT3;2/*  *AtMPT2* | *CsPHT3;5* | Csa04g034060.1 | 104780706 | Chr4: 15804679–15806859 | 1125 | 374 | 40.1 | 9.3 | 4 |
|  | *CsPHT3;6* | Csa06g022670.1 | 104791107 | Chr6: 12755126–12758457 | 1125 | 374 | 40.4 | 9.3 | 4 |
| *AtPHT3;3/*  *AtMPT1* | *CsPHT3;7* | Csa19g054110.1 | 104767030 | Chr19: 23808451–23811023 | 930 | 309 | 34.3 | 9.8 | 4 |
|  | *CsPHT3;8* | Csa15g074360.1 | 104747536 | Chr15: 25733847–25736413 | 930 | 309 | 34.3 | 9.8 | 4 |
|  | *CsPHT3;9* | Csa01g032600.1 | 104791205 | Chr1: 15017664–15019959 | 930 | 309 | 34.3 | 9.8 | 4 |
| *AtPHT4;1* | *CsPHT4;1* | Csa07g014740.1 | 104700536 | Chr7: 6596878–6599540 | 1530 | 509 | 56.2 | 9.1 | 11 |
|  | *CsPHT4;2* | Csa16g015150.1 | 104749907 | Chr16: 5712426–5715050 | 1527 | 508 | 56.3 | 9.1 | 11 |
|  | *CsPHT4;3* | Csa05g034250.1 | 104786692 | Chr5: 12660802–12665429 | 1530 | 509 | 56.4 | 9.1 | 11 |
| *AtPHT4;2* | *CsPHT4;4* | Csa06g040800.1 | 104792601 | Chr6: 20643470–20647116 | 1530 | 509 | 55.1 | 9.7 | 11 |
|  | *CsPHT4;5* | Csa05g015490.1 | 104785671 | Chr5: 5620936–5628866 | 1530 | 509 | 55.0 | 9.4 | 11 |
|  | *CsPHT4;6* | Csa04g051740.1 | 104782239 | Chr4: 24496040–24499719 | 1530 | 509 | 55.0 | 9.7 | 11 |
| *AtPHT4;3* | *CsPHT4;7* | Csa09g045340.1 | 104711167 | Chr9: 16771586–16774486 | 1605 | 534 | 58.2 | 9.7 | 11 |
|  | *CsPHT4;8* | Csa06g020780.1 | 104790922 | Chr6: 11456453–11460007 | 1593 | 530 | 57.9 | 9.8 | 11 |
|  | *CsPHT4;9* | Csa04g029850.2 | 104780506 | Chr4: 14149855–14152770 | 1593 | 530 | 57.7 | 9.7 | 11 |
| *AtPHT4;4* | *CsPHT4;10* | Csa08g001480.1 | 104705300 | Chr8: Chr8: 230348–234133 | 1677 | 558 | 61.6 | 9.2 | 10 |
|  | *CsPHT4;11* | Csa13g057010.1 | 104737737 | Chr13: 23830468–23833909 | 1653 | 550 | 60.7 | 9.1 | 10 |
|  | *CsPHT4;12* | Csa02g001450.1 | 104711898 | Chr2: 240859–244538 | 1665 | 554 | 61.1 | 9.3 | 10 |
|  | *CsPHT4;13* | Csa02g007870.2 | 104715269 | Chr2: 2416948–2420869 | 1101 | 366 | 40.7 | 8.9 | 8 |
| *AtPHT4;5* | *CsPHT4;14* | Csa13g023330.1 | 104736113 | Chr13: 9052413–9057019 | 1578 | 525 | 57.4 | 6.2 | 11 |
|  | *CsPHT4;15* | Csa08g014220.1 | 104706386 | Chr8: 5926518–5931342 | 1578 | 525 | 57.6 | 6.4 | 11 |
|  | *CsPHT4;16* | Csa20g032330.1 | 104770418 | Chr20: 10882188–10886838 | 1584 | 527 | 57.7 | 6.2 | 11 |
| *AtPHT4;6* | *CsPHT4;17* | Csa11g070280.1 | 104725004 | Chr11: 33280932–33283587 | 1299 | 432 | 47.1 | 9.4 | 12 |
|  | *CsPHT4;18* | Csa20g071480.1 | 104771597 | Chr20: 24417095–24419891 | 1299 | 432 | 47.1 | 9.4 | 12 |
|  | *CsPHT4;19* | Csa18g009330.1 | 104760505 | Chr18: 4373340–4375925 | 1299 | 432 | 47.1 | 9.4 | 12 |
| *AtPHT5;1/*  *AtVPT1* | *CsPHT5;1* | Csa07g064340.1 | 104703914 | Chr7: 32705126–32709086 | 2115 | 704 | 78.8 | 6.5 | 9 |
|  | *CsPHT5;2* | Csa09g097720.1 | 104714646 | Chr9: 37194004–37198896 | 2100 | 699 | 78.3 | 6.5 | 9 |
|  | *CsPHT5;3* | Csa16g055720.1 | 104752965 | Chr16: 28162322–28168131 | 1905 | 634 | 70.4 | 5.5 | 9 |
| *AtPHT5;2/*  *AtVPT2* | *CsPHT5;4* | Csa13g049020.1 | 104737184 | Chr13: 19601591–19605453 | 2097 | 698 | 78.3 | 5.8 | 9 |
|  | *CsPHT5;5* | Csa08g044110.1 | 104707475 | Chr8: 18589381–18594544 | 2097 | 698 | 78.3 | 6.0 | 9 |
|  | *CsPHT5;6* | Csa02g016250.1 | 104748770 | Chr2: 5559105–5561998 | 1938 | 645 | 71.8 | 5.2 | 9 |
| *AtPHT5;3/*  *AtVPT3* | *CsPHT5;7* | Csa10g020090.1 | 104718005 | Chr10: 8699068–8702514 | 2103 | 700 | 78.5 | 6.0 | 9 |
|  | *CsPHT5;8* | Csa11g023110.1 | 104722732 | Chr11: 10420297–10423820 | 2103 | 700 | 78.5 | 6.1 | 9 |
|  | *CsPHT5;9* | Csa12g033710.1 | 104731193 | Chr12: 10457959–10461455 | 2103 | 700 | 78.4 | 6.1 | 9 |

**Table S3.** The detailed information on the source of C. sativa seeds.

| **Accession** | **Lot #** | **Country of Origin** | **Plant Name** | **Source** |
| --- | --- | --- | --- | --- |
| PI 650143 | 06ncai01 SD | Germany | CS-CROO | U.S. National Plant Germplasm System |
| - | CASU-1 | Montana | Suneson | The Experimental Farm Network |

**Table S4.** The sequences of primers used for quantitative Real-Time PCR.

| ***C. sativa* Gene** | **Locus ID** | **NCBI Gene ID** | **NCBI Reference Sequence** | **Forward Primer (5′ 🡪 3′)** | **Reverse Primer (5′ 🡪 3′)** | **Size (bp)** |
| --- | --- | --- | --- | --- | --- | --- |
| CsTUA | Csa17g085890.1 | 104758270 | XM_010481098.1 | ACTTGGCTTGCTGTTTGATG | CAGTTGGTGGCTGGTAGTTG | 159 |
| CsPHT1;1 | Csa11g071540.1 | 104725125 | XM_019232427.1 | TGCTTCTTCAGGTTTTGGTTG | AAAGCCCCACGAGTCTTCTT | 101 |
| CsPHT1;12 | Csa20g067290.1 | 104771483 | XM_010496012.2 | GGCCAAGACTGACGCAGGA | TGACTATTTCTCGTCCGGGCT | 175 |
| CsPHT1;28 | Csa03g024720.1 | 104776208 | XM_010500225.1 | GACGTTATAAGAATCATTGTCCTGT | GTATTCGAGCTGCATCAAGCG | 179 |
| CsPHT1;31 | Csa07g047990.1 | 104702405 | XM_010418262.2 | GCTAACAAAAGGACGCGTGG | AAACTCCCTCCTCCGACGTT | 128 |
| CsPHT1;33 | Csa16g040570.1 | 104751540 | XM_010473496.2 | TCTTAGGCCACACTTTCAGTCC | CTGTATCTTTGCGGCGGTGT | 146 |
| CsPHT2;1 | Csa06g006270.1 | 104790285 | XM_010515998.2 | ATTTGTGAGGCCCAACCAGAC | AGGTGGAGGTGAGAGGATTGT | 162 |
| CsPHT3;1 | Csa08g006050.1 | 109124421 | XM_010421723.1 | GCAAGCGTGAGATCGGGCTAT | CACCTGGTCGAGGAGGAAAGA | 147 |
| CsPHT3;7 | Csa19g054110.1 | 104767030 | XM_010491049.2 | AGTAGAAGACCTGAAGCAAGC | TCACTGACAAGAATTGGTTTGCC | 106 |
| CsPHT4;1 | Csa07g014740.1 | 104700536 | XM_010416063.2 | AATCGTCCACCTGAAAAATCCG | CGGACCTGTTCCTCGGGTA | 91 |
| CsPHT4;6 | Csa04g051740.1 | 104782239 | XM_010507114.2 | CACACTACGCCAGTCAGTACA | ACGATGTGAAGGTTAGGAGTGG | 108 |
| CsPHT4;15 | Csa08g014220.1 | 104706386 | XM_010422575.1 | TCAAGGGTTAAGTGCTCCGC | CCCCTTTCCGTTTCCTTACTC | 146 |
| CsPHT4;17 | Csa11g070280.1 | 104725004 | XM_019232187.1 | AAACTCATCTCCTTCACCGTGG | AGATTTGGGGGAAAATGACGGA | 125 |
| CsPHT5;1 | Csa07g064340.1 | 104703914 | XM_010420003.2 | CTACTTTGCCTGGGTGGGTT | AGCAGATTACTTGATGTTGTTTCA | 139 |
| CsPHt5;4 | Csa13g049020.1 | 104737184 | XM_010457294.2 | ACACAATCTCTGGAAATCGGA | GGCGACCATTTTCTTTTGACG | 130 |
| CsPHT5;8 | Csa11g023110.1 | 104722732 | XM_010440947.2 | GTTGCAACTCAGAGAGAAGAAGA | GGCGACGTTCAAGATTCCCT | 158 |
